# Post-Exercise Rehydration: Effect of Consumption of Beer with Varying Alcohol Content on Fluid Balance after Mild Dehydration

**DOI:** 10.3389/fnut.2016.00045

**Published:** 2016-10-17

**Authors:** Annemarthe H. C. Wijnen, Jora Steennis, Milène Catoire, Floris C. Wardenaar, Marco Mensink

**Affiliations:** ^1^Division of Human Nutrition, Wageningen University, Wageningen, Netherlands; ^2^Sports and Exercise studies, HAN University of Applied Sciences, Heyendaalseweg 141, Nijmegen, Netherlands

**Keywords:** rehydration, exercise, alcohol, beer, urine output, fluid balance

## Abstract

**Purpose:**

The effects of moderate beer consumption after physical activity on rehydration and fluid balance are not completely clear. Therefore, in this study, we investigated the effect of beer consumption, with varying alcohol content, on fluid balance after exercise-induced dehydration.

**Methods:**

Eleven healthy males were included in this cross over study (age 24.5 ± 4.7 years, body weight 75.4 ± 3.3 kg, VO_2max_ 58.3 ± 6.4 mL kg min^−1^). Subjects exercised on a cycle ergometer for 45 min at 60% of their maximal power output (*W*_max_) until mild dehydration (1% body mass loss). Thereafter, in random order, one of five experimental beverages was consumed, in an amount equal to 100% of their sweat loss: non-alcoholic beer (0.0%), low-alcohol beer (2.0%), full-strength beer (5.0%), an isotonic sports drink, and water. Fluid balance was assessed up till 5 h after rehydration.

**Results:**

After 1 h, urine production was significantly higher for 5% beer compared to the isotonic sports drink (299 ± 143 vs. 105 ± 67 mL; *p* < 0.01). At the end of the 5-h observation period, net fluid balance (NFB) was negative for all conditions (*p* = 0.681), with the poorest fluid retention percentage for 5% beer (21% fluid retention) and the best percentage for the isotonic sports drink (42%). Non-alcoholic beer, low-alcoholic beer, and water resulted in fluid retention of 36, 36, and 34%, respectively (*p* = 0.460).

**Conclusion:**

There was no difference in NFB between the different beverages. Only a short-lived difference between full-strength beer and the isotonic sports drink in urine output and NFB was observed after mild exercise-induced dehydration. Fluid replacement – either in the form of non-alcoholic beer, low-alcoholic beer, full-strength beer, water, or an isotonic sports drink of 100% of body mass loss was not sufficient to achieve full rehydration. The combination of a moderate amount of beer, with varying alcohol content, enough water or electrolyte- and carbohydrate beverages, and salty foods might improve rehydration, but more research is needed.

## Introduction

Adequate rehydration after exercise is important. If not appropriately replaced, dehydration may affect performance, especially when there is little time in between sports events (1). It is not always possible to drink enough fluids during exercise (2). Therefore, many active people choose to replace their fluid losses afterwards. According to the American College of Sports Medicine (ACSM), an optimal rehydration beverage consists of water, carbohydrates, sodium, and potassium (3). Glucose- and electrolyte sports drinks are effective rehydration beverages, but other drinks, such as milk, also sufficed as rehydration beverages in multiple studies (4, 5).

A beverage regularly consumed after exercise among team-based sports is beer (6, 7). Beer contains carbohydrates, water, and small amounts of sodium and potassium, but also alcohol. Alcohol is known to increase urine output (8, 9), which could interfere with adequate rehydration after exercise. Some studies specifically examined the effect of alcohol on net fluid balance (NFB) after exercise and suggest that the diuretic effect of alcohol (alcohol content ≤4%) is blurred when the body is dehydrated (10, 11). Other research showed that consumption of a large amount of full-strength beer does increase urine output (12). The amount of alcohol in beer seems to influence the rehydration capacity of beer.

Despite the fact that beer contains alcohol, its other components might have a favorable influence on NFB after extensive exercise, such as sodium and carbohydrates. Sodium helps retaining ingested fluids, stimulates thirst and restores lost plasma volume more rapidly (3). However, the amount of sodium in beer appears to be too low to have an effect on NFB (11). Carbohydrates accelerate water absorption in the small intestine by stimulating carrier-mediated water movement (13), but are even more important to refill depleted glycogen stores after exercise (14).

Previous studies addressing beer and rehydration used rather extreme protocols, where subjects received a large amount of alcohol, up to 2.5 L of beverages containing 4–4.8% alcohol (11, 12, 15, 16). The amount of alcohol consumed was much higher in these studies than the general alcohol guidelines of ~2 alcoholic consumptions per day for men. Most of these studies (11, 15, 16) followed the recommendation to consume a fluid volume of 150% to replenish fluid losses (3), which might not be compatible with drinking a moderate amount of alcoholic beverages. A recent Spanish study (17) examined the effect of a more moderate amount of beer (4.5%) of 660 mL. However, in this study participants also consumed *ad libitum* water, making it difficult to determine the effect of the beer on NFB. Furthermore, in all the studies so far, the impact of the alcoholic beverages was not compared with typical post-exercise rehydration drinks.

Therefore, the objective of this study was to assess the effect of beer in moderate amounts with varying alcohol content on NFB after exercise-induced dehydration. The rehydration capacity of these beverages was compared with a rehydration drink and water. In order to increase the applied value of the present research, we selected beverages that are commonly available in the sports canteen.

## Materials and Methods

### Participants

Thirteen healthy males volunteered in this study, but two participants were excluded from the final analyses, as they did not complete the study due to illness (influenza). All were habitually physically active and exercised one to four times a week. They drank beer occasionally (defined as ≤14 consumptions per week), and had no (family) history of alcoholism. They did not use any drugs or medication that could influence NFB.

The study was approved by the Medical Ethical Committee of Wageningen University. All participants provided written informed consent after they received oral and written information about the study.

### Sample Size Calculation

Based on data of Shirreffs and Maughan (11), we calculated that ~80% of fluid losses could be recovered with the non-alcoholic, low-alcohol, and water conditions, resulting in a urine output of ~150 mL. For full-strength beer, we assumed ~60% of fluid losses would be recovered with a urine output of ~300 mL. Thus, a difference of ~150 mL in urine output was expected between the conditions. Urine output can vary greatly between individuals (10, 11); therefore, we expected the SD to be equal to the expected difference of 150 mL. With a two-sided significance of 0.05 and power of 0.80, it was estimated that eight participants would be needed to detect differences in urine output between the groups. We aimed to recruit 12 participants.

### Experimental Design

The study had a randomized crossover design. Each participant performed five experimental trials of exercise-induced mild dehydration (1% body mass loss), followed by beverage ingestion and a 5 h observation phase. The trials were separated by a period of at least 7 days. The participants received a compensation of € 180 for participation in this study.

Beverage types varied over the five trials. Participants were randomly exposed to the following: non-alcoholic beer (0.0%, Amstel 0.0^®^, Heineken, The Netherlands), low-alcohol beer (2.0%, Lingen’s Blond^®^, Heineken, The Netherlands), pilsner (5.0%, Amstel^®^ Pilsner, Heineken, The Netherlands), tap water, or a commercially available isotonic glucose–electrolyte rehydration drink (sports drink, Isostar Hydrate & Perform lemon^®^, Nutrition et Sante, France) (see Table 1 for nutritional details). The beers were all purchased at the same time and came from the same batch of production to avoid possible differences in ingredients and nutritional composition.

**Table 1:** **Nutritional values of the beverages**.

	Energy (kJ)^a^	Carbohydrates (g)^a^	Sodium (mmol L^−1^)	Alcohol (% volume)
0% beer	104	6	1.3	0.0
2% beer	125	4	0.6	2.0
5% beer	167	3	1.1	5.0
Sports drink	125	7	29.6	0.0
Water	0	0	0.8	0.0

*^a^Presented as 100 g of product*.

Total duration of the study for each participant was six weeks. About 2 weeks before the start of the first experimental trial, a preliminary test was performed, to measure maximal aerobic capacity (VO_2max_) and *W*_max_. After a short warm up, participants performed an incremental aerobic exercise test on a cycle ergometer during which breath-by-breath gas samples were collected (Oxycon Pro™, Carefusion Corporation & Lode Excalibur, Lode B.V., Groningen, The Netherlands). VO_2max_ was defined as the highest VO_2_ during any 30 s in any given period during the exercise test. The highest wattage participants reached during the test was determined as *W*_max_.

Participants received a diary in which they kept track of any signs of illness, deviations from the protocol, or any side effects throughout the duration of the study.

### Experimental Protocol

The day before each trial, participants were asked to drink at least 2 L of fluid, refrain from alcohol and strenuous exercise. Participants were fasted from 10:00 p.m. the night before each test day. They used the same mode of transport to travel to the university on all test days.

Each test day started with a morning urine collection at their home. After arriving at the university early in the morning, they ate a standard breakfast: one brown bun, one currant bun, a slice of gingerbread, two sweet, low-fat bread toppings, and one glass of water (150 mL). The breakfast provided ~1348 kJ, 2 g total fat, 6 g protein, 68 g carbohydrates, and 279 mg sodium. This was calculated with Eetmeter software version August 2016 (Het Voedingscentrum, The Hague, The Netherland). Afterwards participants collected urine for the second time and their semi-nude bodyweight was measured on a calibrated scale (SECA 877, Vogel & Halke GmbH & Co KG, Hamburg, Germany). In order to lose 1% of their initial bodyweight, participants cycled at 60% of their pre-determined *W*_max_. During exercise, room temperature was increased until 23–25°C to accelerate sweat loss. Humidity was not measured. Semi-nude towel-dry bodyweight was measured at a 10-min interval during exercise to determine weight loss. After losing 0.80% of their initial bodyweight, they stopped exercising, took a shower and had to empty their bladder. Next, semi-nude towel-dry bodyweight was measured for the second time to calculate total body mass loss.

In the next 45 min, the participants received the experimental beverage in an amount equivalent to 100% of their body mass loss. To account for urinary fluid losses, the American College of Sports Medicine (ACSM) recommends drinking a volume equivalent to 150% of fluid loss to rehydrate after exercise (3). Due to ethical constraints and the choice to examine moderate alcohol consumption, we did not adhere to this guideline. Ethanol intake was calculated by multiplying fluid intake in milliliter times alcohol percentage (2% for low-alcoholic beer and 5% for full-strength beer) and again multiplied by the density of alcohol (0.789 g mL^−1^). The beverage was provided in three equal portions, 15 min apart, in the same cups. Even though no information was given on beverage type, all participants were able to guess which drink they consumed. After consumption of the last portion of the beverages (*t* = 0 h), the participants remained rested (sitting, reading, working) and were not allowed to ingest any fluid and food for the next 5 h. At time point 0, 1, 2, 3, and 5 h, urine was collected. The participants were asked to empty their bladder completely at each voiding.

After 3 and 5 h in the post-rehydration phase, breath alcohol content (BrAC) was measured (measure range: 0–0.500 BrAC%, Alcotest 6510, Dräger Safety AG & CO. KGaA, Lübeck, Germany) to make sure the participants could go home safely. When BrAC was ≤0.22 mg L^−1^ expired air after 5 h, participants were allowed to leave the research facilities. Each test day ended with a lunch.

### Urine Collection and Analysis

Each urine sample was accurately weighed to the nearest gram on a calibrated scale (Sartorius 1203 MP, Sartorius AG, Göttingen, Germany), and an aliquot (5 mL) was taken and stored at −20°C in a freezer until further analysis. Urine osmolality was measured using freezing-point depression (Osmomat 030, automatic cryoscopic osmometer, Gonotec, Berlin, Germany). Sodium and potassium concentrations were measured by V-Lyte IMT (Dimension Vista 1500, Siemens Healthcare Global, Erlangen, Germany).

Urine output at *t* = 0, 1, 2, 3, and 5 h after rehydration, body mass change, and fluid intake were used to calculate NFB. NFB was defined as follows: fluid intake – sweat loss – urine output. Sweat loss was estimated from exercise-induced body mass loss. Percentages of fluid retention were calculated by dividing retained fluid by ingested fluid.

### Statistical Analysis

Data are presented as mean ± SD. All data were normally distributed. Changes in body mass, rehydration percentage, and urine output were analyzed by one-way repeated measures analysis of variance (ANOVA). Two-way repeated measures ANOVA was used to identify differences between trials and changes over time for cumulative urine output. When sphericity could not be assumed, the degrees of freedom were corrected with the Greenhouse-Geisser correction. Urine sodium, potassium, and urine osmolality were analyzed using linear mixed models, due to missing values in the dataset. *Post hoc* analyses were performed for any significant main effect (*P* < 0.05) with a Bonferroni adjustment. All statistical analyses were carried out using IBM SPSS Statistics, v23 (Chicago, IL, USA).

## Results

### Participants

Mean age was 24.5 ± 4.7 years, weight 75.4 ± 3.3 kg, and BMI 22.4 ± 1.8 kg m^−2^. VO_2max_ averaged 58.3 ± 6.4 mL kg min^−1^, indicating that the participants (*N* = 11) were well trained. The average workload of the experimental trials was 210 ± 30 W. No signs of illness, deviations from the protocol, or side effects were reported.

### Pre-Hydration Phase

Pre-exercise body mass did not differ between the trials [all averaging 75.1 ± 4 kg (*p* = 0.692)]. This also applied to pre-exercise urine osmolality [see Table 2 (first morning *p* = 0.937 and pre-exercise *p* = 0.564)], showing that participants were in a similar state of hydration before exercise.

**Table 2:** **Urine osmolality**.

Trial	First morning	Pre-exercise	Post-exercise	Time after rehydration (h)				
				0	1	2	3	5
0% beer	543 ± 279	655 ± 215	749 ± 128	779 ± 278	458 ± 309	557 ± 227	540 ± 280	625 ± 326
2% beer	561 ± 247	603 ± 264	634 ± 178	731 ± 289	355 ± 291	490 ± 227	543 ± 219	567 ± 277
5% beer	539 ± 148	660 ± 164	784 ± 140	766 ± 87	206 ± 63^a^	459 ± 223	643 ± 189	656 ± 252
Sports drink	502 ± 181	557 ± 255	747 ± 166	775 ± 273	584 ± 249	550 ± 296	571 ± 207	546 ± 258
Water	578 ± 183	517 ± 280	665 ± 272	728 ± 274	461 ± 264	492 ± 298	554 ± 267	709 ± 201
*p*-Value	0.940	0.560	0.286	0.983	0.014	0.880	0.837	0.605

*Data are presented as mean ± SD and expressed in mOsmol kg^−1^*.

*^a^Significantly different from sports drink (*post hoc* test *p* < 0.05)*.

Exercise-induced body mass loss and, hence, level of dehydration did not differ between conditions, and averaged 0.99 ± 0.14% over all trials (0.95 ± 0.10%, 1.04 ± 0.15%, 0.98 ± 0.09%, 0.99 ± 0.16%, and 0.98 ± 0.20% for 0, 2, 5% beer, sports drink, and water, respectively; *p* = 0.437). Mean exercise time was the same in each trial [44 ± 1 min, 44 ± 1 min, 45 ± 2 min, 45 ± 1 min, and 45 ± 1 min (*p* = 0.09)].

### Rehydration Phase

The ingested volume of fluid for rehydration after exercise was equal for all trials (mean 736 ± 105 mL, *p* = 0.748). The mean alcohol intake was 12.1 ± 1.6 g for 2% beer and 28.9 ± 2.8 g for 5% beer.

### Post-Rehydration Phase

#### Urine Output and Fluid Balance

**Figure 1:**
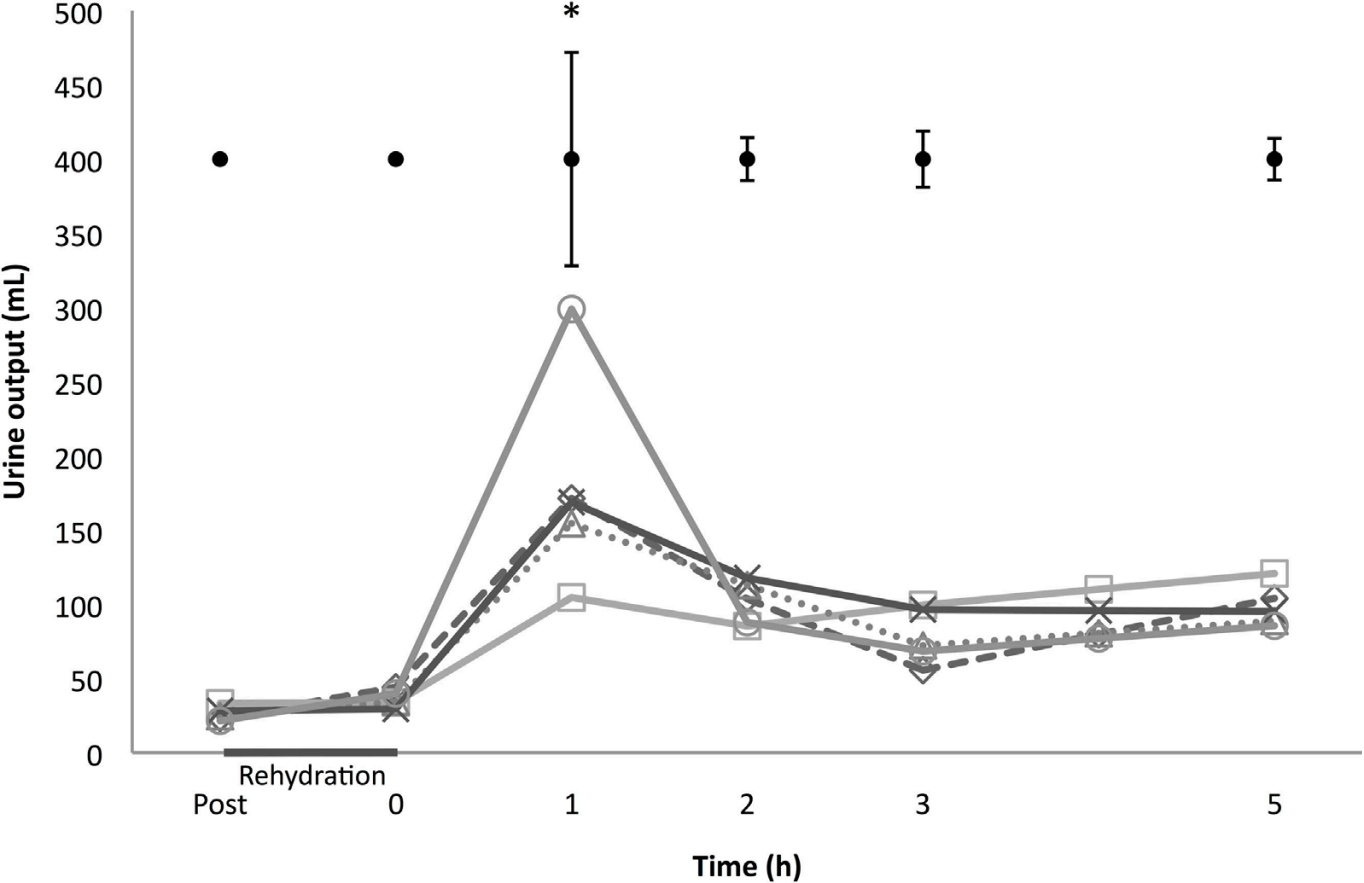
**Urine output**. Starting post-exercise and ending 5 h after rehydration: Δ 0% beer; × 2% beer; ○ 5% beer; □ sports drink; ◊ water. At the top are ±1 pooled SD bars per time point. *p < 0.01 between 5% beer and sports drink, 1 h in the post-rehydration phase.

There was a significant effect of time and time × treatment on urine output (*p* < 0.001; Figure 1). Peak urine production occurred 1 h after rehydration for all trials. The highest urine volume at this time point was seen with 5% beer, the lowest in the sports drink condition (299 ± 143 mL vs. 105 ± 67 mL; *post hoc p* < 0.01). Cumulative urine output over the entire 5 h post-rehydration phase did not differ between conditions (*p* = 0.681; Table 3).

**Table 3:** **Cumulative urine output**.

Trial	Time after rehydration (h)				
	0	1	2	3	5
0% beer	34 ± 24	189 ± 151	301 ± 202	374 ± 222	462 ± 241
2% beer	30 ± 14	198 ± 135	316 ± 203	412 ± 245	507 ± 288
5% beer	40 ± 17	339 ± 152^a^	426 ± 168	495 ± 171	580 ± 188
Sports drink	34 ± 27	139 ± 75	223 ± 132	324 ± 177	444 ± 240
Water	44 ± 29	216 ± 39	319 ± 187	374 ± 202	478 ± 202
*p*-Value	0.619	0.012	0.151	0.386	0.682

*Data are presented as mean ± SD and expressed in milliliter*.

*^a^Significantly different from sports drink (*post hoc* test *p* < 0.05)*.

**Figure 2:**
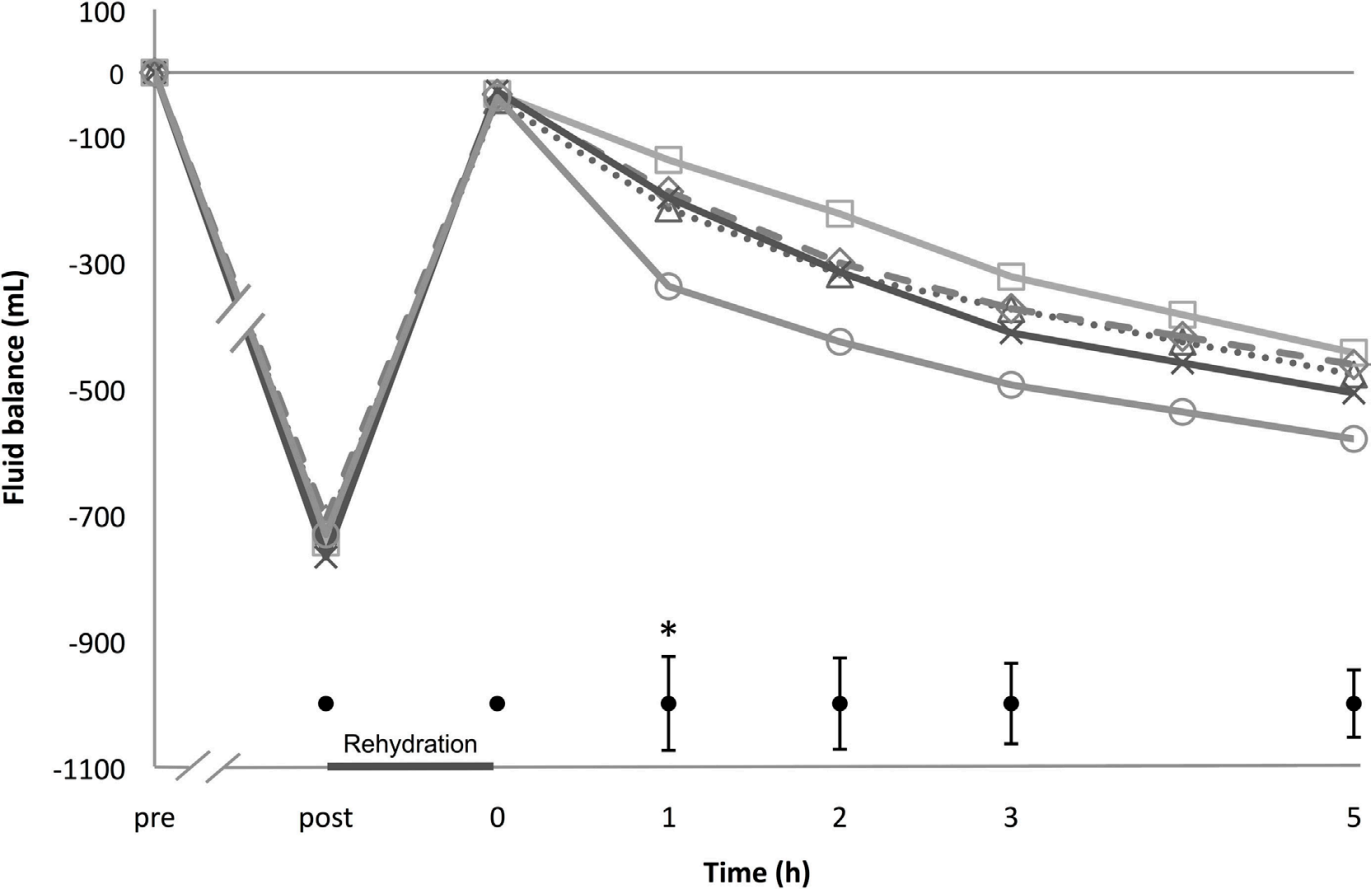
**Net fluid balance**. Starting pre-exercise and ending 5 h after rehydration; Δ 0% beer; × 2% beer; ○ 5% beer; □ sports drink; ◊ water. At the bottom are ± 1 pooled SD bars per time point. *p < 0.05 between 5% beer and sports drink, 1 h in the post-rehydration phase.

Cumulative whole body NFB is depicted in Figure 2. A clear time effect was observed (*p* < 0.001), but no treatment (*p* = 0.104) or interaction effect (*p* = 0.167). One hour after rehydration, NFB differed significantly between the 5% beer and sports drink condition (Figure 2; *post hoc p* < 0.01).

At the end of the 5 h post-rehydration phase, fluid retention percentages were 36.3 ± 29.9% for 0% beer, 35.7 ± 31.0% for 2% beer, 21.1 ± 24.2% 5% beer, 33.9 ± 25.4% for water, and 42.2 ± 22.9% for sports drink, respectively. There was no statistically significant difference between the beverages (*p* = 0.460).

#### Urine Composition

In Table 2, urine osmolality is shown. Post-exercise urine osmolality was increased for all conditions, and increased even further directly after the rehydration phase (*t* = 0 h). One hour after rehydration urine osmolality decreased and reached its lowest level, thereafter increasing until the end of the post-rehydration phase (*p* = 0.001). The 5% beer condition had the lowest osmolality value after 1 h, which differed significantly from the sports drink condition (*p* < 0.05). No clear treatment effect was observed for the entire post-rehydration phase (*p* = 0.855), but there was a significant interaction effect (*p* < 0.005).

Urine sodium and potassium concentration changed over time (both *p* < 0.001), but did not differ between the trials (*p* = 0.927 and *p* = 0.633). One hour after rehydration urine potassium differed between conditions (*p* = 0.010), with a significant lower amount for 5% beer than the sports drink condition (*post hoc p* < 0.05). The same observation was made for urine sodium (*p* = 0.032). After drinking 5% beer the amount of urine sodium was significantly lower than after the sports drink condition 1 h after rehydration (*post hoc p* < 0.05). Urine sodium and potassium levels per hour can be found in Table 4.

**Table 4:** **Urine potassium and sodium in the post-rehydration phase**.

	Time after rehydration (h)	0%	2%	5%	Sports drink	Water	*p*-Value
Sodium	0	79 ± 52	90 ± 54	73 ± 36	87 ± 51	72 ± 53	0.876
	1	59 ± 38	56 ± 39	34 ± 14^a^	71 ± 43	86 ± 44	0.032
	2	68 ± 30	69 ± 28	65 ± 37	62 ± 46	66 ± 40	0.991
	3	52 ± 29	57 ± 23	74 ± 35	56 ± 41	69 ± 43	0.555
	5	72 ± 36	81 ± 39	72 ± 38	80 ± 37	70 ± 48	0.952
Potassium	0	48 ± 31	46 ± 20	50 ± 16	58 ± 37	56 ± 37	0.857
	1	25 ± 20	16 ± 12	11 ± 10^a^	38 ± 31	38 ± 24	0.010
	2	45 ± 19	43 ± 23	31 ± 20	57 ± 45	61 ± 42	0.220
	3	53 ± 25	53 ± 26	53 ± 26	56 ± 31	58 ± 30	0.994
	5	51 ± 33	53 ± 27	64 ± 30	57 ± 32	44 ± 24	0.622

*Data are presented as mean ± SD and expressed in mmol L^−1^*.

*^a^Significantly different from sports drink (*post hoc* test *p* < 0.05)*.

### Breath Alcohol Concentration

Breath alcohol content was 0.0 ± 0.0 mg L^−1^ for all participants 3 and 5 h in the post-rehydration phase. Therefore, they could leave the research facilities immediately after they had eaten their lunch.

## Discussion

Our results suggest that after mild exercise-induced dehydration, a moderate amount of non-alcoholic beer, low-alcoholic beer, full-strength beer, water, or an isotonic sports drink all fail to replenish depleted fluid losses to the same extent. Full-strength beer had the tendency to increase urine output as there was a short-lived difference in urine output between full-strength beer and the sports drink 1 h after rehydration. Overall, there were no significant differences in NFB between the beverages, although retention fraction was twice as high for the sports drink compared to 5% beer, while low-alcoholic beer behaved comparable to water and non-alcoholic beer.

Drawing a comparison between our study and previous work is difficult because of the variety in research protocols. There were, for example, differences in alcohol percentages, sodium and potassium content of the beverages, level of dehydration, and monitoring time after rehydration (6, 7, 10–13). A summary of characteristics of previous research is provided in Table 5. Despite the differences in research protocols, previous studies concur with our results, showing full-strength beer only had the tendency for increased urine output when dehydrated (10, 11). Non-alcoholic beer, low-alcoholic beer, and water turned out to have similar rehydration percentages and were also not associated with an increased urine output, confirming earlier observations (10–12, 17).

**Table 5:** **Summary of randomized controlled crossover studies**.

Reference	Total *n*^a^	Treatment	Exercise-induced BM loss (%)^b^	Fluid volume intake for rehydration (%)	Fluid intake (L)^b^	Alcohol intake (g)	Monitoring time (h)	Effect on cumulative urine output (*p*)	Euhydration after treatment?
Shirreffs and Maughan (11)	6	0% beer	2.0	150	2.2	0.0	6	0.307	Only <1 h after treatment
		1% beer^c^				17.7			
		2% beer^c^				35.9			
		4% beer^c^				68.0			
Hobson and Maughan (10)	12	Euhydrated + 0% beer	Euhydrated: ~0.6	-	1.0	0.0	4	0.057	-
		Euhydrated + 4% beer^c^				31.6		<0.001	
		Dehydrated + 0% beer	Dehydrated: ~2.5		Equal amount for all treatments	0.0		Higher urine output with euhydrated treatments vs. dehydrated.	
		Dehydrated + 4% beer^c^				31.6			
Desbrow et al. (15)	7	2.3% beer	2.0	150	2.5	57^d^	4	<0.05^e^	Only <1 h after treatment
		2.3% beer + 25 mmol L^−1^ Na				59^d^			
		4.8% beer				120^d^		Lower urine output for 2.3% + 25 vs. 4.8 and 4.8% + 25	
		4.8% beer + 25 mmol L^−1^ Na				122^d^			
Flores-Salamanca and Aragón-Vargas (12)	11	0.5% beer	2.1	100	1.6	0.0	3	0.001	No
		4.5% beer				6.4		Higher urine output for 4.5 vs. 0.5% and water	
		Water				60.1			
Jiménez-Pavón et al. (17)	16	660 mL 4.5% beer + mineral water *ad libitum*	~2.4	~90	1.6	~24	–	0.70	No
		Mineral water *ad libitum*				0.0			Rehydration period was 2 h, no separate monitoring period
Desbrow et al. (16)	12	2.3% beer + 25 mmol L^−1^ Na	2.0	150	2.3	~53^d^	4	<0.05^e^	Only <1 h after treatment
		2.3% beer + 50 mmol L^−1^ Na				~52^d^			
		3.5% beer + 25 mmol L^−1^ Na				~81^d^		Lowest urine output for 2.3% + 50 vs. all other treatments	
		3.5% beer + 50 mmol L^−1^ Na				~78^d^			
Wijnen, present study	11	0% beer	1.0	100	0.74	0.0	5	0.681	No
		2% beer				12.1			
		5% beer							
		Isotonic sports Drink				28.9			
		Water							

*^a^All participants were males*.

*^b^Data have been rounded up to the nearest tenth*.

*^c^These beverages consisted of a non-alcoholic base with alcohol added to it*.

*^d^Differences in g alcohol in beverages with the same alcohol percentage occurred because of small differences in body mass loss*.

*^e^Exact significant p-value or F ratio for main effects was not reported*.

One previous study did find a negative effect of full-strength beer on NFB (12). In that study participants had a significantly higher urine output and a more negative NFB after drinking full-strength beer compared to low-alcoholic beer and water. The level of dehydration and alcohol intake were twice as high as in the present study, as depicted in Table 5. Therefore, our study protocol might not have been extreme enough to detect significant differences. And even though we included more participants than required according to our calculations, sample size may have been too small. The calculations were based upon a study with a higher alcohol intake and higher level of hypohydration (11) and may, therefore, have caused an underestimation of how many participants were needed.

Rehydration drinks typically consist of water, carbohydrates, and electrolytes. Carbohydrate content of beer, in particular of non-alcoholic beer, is close to what is found in isotonic rehydration drinks (60–80 g L^−1^). Sodium is even more important for rehydration. The ACSM (3) defined ~20–30 mmol L^−1^ as an optimal amount of sodium for rehydration beverages. Shirreffs and Maughan (18) demonstrated that participants retained a mean of 71% of the ingested volume when the beverage had a high sodium concentration (102 mmol L^−1^) but only 37% when virtually no sodium was present (1 mmol L^−1^). The sodium content of our beers (as shown in Table 1) clearly limited the rehydration capacity: 36, 36, and 21% after 5 h for 0% beer, 2% beer, and 5% beer, respectively. Indeed, it was shown that addition of 25–50 mmol L^−1^ of sodium to a low-alcohol beer (2.3% alcohol) significantly reduced urine output compared with low-alcohol beer without sodium. When 25 mmol L^−1^ sodium was added to full-strength beer (4.8% alcohol) urine output remained high compared to low-alcoholic beer with 2.3% alcohol (15, 16). This indicates that when in a dehydrated state, alcohol-induced diuresis is more prominent than the effects of sodium in that condition. The commercially available sports drink in our study contained an optimal amount of sodium: 29.6 mmol L^−1^, resulting in the highest, but non-significantly different, rehydration capacity of 42% compared to 0, 2 and 5% beer and water.

The mechanisms through which alcohol affects diuresis are still not completely clear. Arginine vasopressine (AVP) plays an important role in hydration status, preventing swings in fluid balance to avoid de- and hyperhydration. When dehydrated the level of AVP increases, triggered by increasing blood osmolality. Alcohol suppresses AVP, independently of osmolality, resulting in an increased urine output (9, 19). The duration of the presence of alcohol in the body and the level of dehydration might influence which of these mechanisms prevails (10, 20). However, studies that measured AVP levels (11, 16) did not find any differences after ingestion of non-alcoholic or beer containing alcohol. Unfortunately, we did not collect blood samples to assess AVP, aldosterone, plasma osmolality, blood alcohol concentration, alcohol dehydrogenases, and other relevant markers to further explore this topic.

Another point for future research should be to examine the effects of moderate alcohol consumption on rehydration after exercise in women, as all research until now focused on men. Alcohol likely affects fluid balance differently in women because of differences in body composition (e.g., lower body mass and higher fat mass compared to men). Furthermore, the menstrual cycle can influence body water status (21) and sweat rates are generally lower in women due to lower body mass. Rehydration strategies may, therefore, be different in women (3).

We deliberately chose a more applicable and applied approach in our study as compared to previous research on this topic: dehydration was limited to 1% of body weight, and fluid replacement was 100% of the calculated sweat loss. Although traditional post-exercise drinking habits might be different in some sports, this amount is closer to recommendations regarding alcohol intake for the male adult population and, therefore, less detrimental to overall health. Moreover, all investigated beverages are commonly available in the sports canteen, making the conclusion of this study applicable in practice. However, euhydration was never achieved in our study because subjects were rehydrated with a volume of 100% of fluid loss instead of 150% to account for extra urinary losses (3). Previous research showed that even when full-strength beer was consumed in a volume of 150% of fluid loss and with the addition of extra sodium one still ends up dehydrated (15, 16). This confirms that the guidelines for hydration and for moderate alcohol consumption are not compatible. One study showed that a higher rehydration percentage can be achieved (rehydration ~80%) when beer is consumed in combination with *ad libitum* water within 2 h after exercise. This study did not, however, report follow-up time (17). Alternatively, addition of sodium to low-alcoholic beer might significantly reduce urine output, but a high amount of 50 mmol L^−1^ can negatively affect palatability (16). From a practical point of view, drinking a moderate amount of either non-, low-alcoholic or maybe even full-strength beer in combination with enough water and eating some salty snacks could overcome the alcohol-induced diuresis and improve rehydration, but more research is needed. It should also be noted that heavy alcohol consumption influences other factors in post-exercise recovery, but moderate amounts of 0.5 g alcohol kg^−1^ bodyweight have been shown not to impact glycogen recovery and muscle injury (22). When one chooses to drink alcohol, moderation is key.

In conclusion, there was only a short-lived difference between full-strength beer and the isotonic sports drink in urine output and NFB. Although no overall differences in NFB between the different beverages were observed, retention fraction was twice as high for the sports drink compared to full-strength beer, while low-alcoholic beer, non-alcoholic beer, and water did not differ. Also after mild exercise-induced dehydration, fluid replacement – either in the form of non-alcoholic beer, low-alcoholic beer, full-strength beer, water, or an isotonic sports drink of 100% of body mass loss – is not sufficient to achieve full rehydration. The fluid volume was too low to comply with rehydration guidelines of 150% and our sample size might have been too small to detect any differences among beverages, but previous research showed a higher amount of full-strength beer might not improve rehydration (15, 16). Therefore, further research is needed to determine how to achieve euhydration with moderate beer consumption, varying in alcohol content, in combination with water, glucose- and electrolyte beverages, and salty foods to achieve euhydration after exercise.

## Author Contributions

The study was designed by FW and MM. Data were collected by AW and JS. The interpretation of data and preparation of the manuscript were done by AW and MM. MC supervised the study together with MM.

## Conflict of Interest Statement

There was no conflict of interest during the design, performance, data analysis and writing of the publication of this study. All authors were affiliated to Wageningen University at that time. Only during the submission process AW was employed at the Dutch Beer Institute. The other authors (JS, MC, FW, MM) do not have a conflict of interest.
